# Whole-genome sequencing of *Enterococcus faecalis* probiotic strains isolated from raw milk of healthy cows

**DOI:** 10.1128/mra.00465-24

**Published:** 2024-08-20

**Authors:** Md. Morshedur Rahman, Naim Siddique, ANM Aminoor Rahman, Ziban Chandra Das, Tofazzal Islam, M. Nazmul Hoque

**Affiliations:** 1 Molecular Biology and Bioinformatics Laboratory, Department of Gynaecology, Obstetrics and Reproductive Health, Bangabandhu Sheikh Mujibur Rahman Agricultural University (BSMRAU), Gazipur, Bangladesh; 2 Institute of Biotechnology and Genetic Engineering (IBGE), BSMRAU, Gazipur, Bangladesh; University of Maryland School of Medicine, Baltimore, Maryland, USA

**Keywords:** whole genomes, *Enterococcus faecalis*, probiotics, cow, raw milk

## Abstract

We report the draft genomes of two *Enterococcus faecalis* strains MBBL1 and MBBL2, isolated from raw milk of healthy cows. The genome of MBBL1 is 2,681,695 bp with 57.41× coverage, and MBBL2 is 2,681,119 bp with 99.81× coverage, spanned across 14 and 13 contigs, respectively.

## ANNOUNCEMENT

*Enterococci*, a type of lactic acid bacteria (LAB), are commonly found in the gut of humans and animals (1). Like most other LABs, *E. faecalis* is used as a probiotic to maintain healthy gastrointestinal microbiota and reduce gastrointestinal inflammation (1–3). Recently, *E. faecalis* has gained notable interest due to its therapeutic prospects (2). They also demonstrate the ability to produce bacteriocins or bacteriocin-like substances (BLISs), preventing harmful bacteria colonization and regulating gut microbes to protect against pathogens (1, 4, 5). Due to the antimicrobial activity against pathogens and its innocuous nature toward all other LAB, *E. faecalis* can emerge as a novel probiotic candidate (2, 6, 7).

We isolated *E. faecalis* strains from healthy cow’s milk collected from the BSMRAU dairy farm (24.09° N, 90.41° E), Gazipur, Bangladesh. We performed the California Mastitis Test (CMT) to ensure the milk was free from mastitis (8). Milk samples were cultured in MRS (deMan, Rogosa, and Sharpe) broth (HiMedia, India) for overnight incubation at 37°C. Subsequently, inoculum was streaked onto MRS agar plates for 36- to 48-h incubation at 37°C (5). Phenotypic identification of the isolates was carried out on the basis of colony morphology, Gram staining (Gram-positive cocci), and biochemical tests (i.e., indole, Voges-Proskauer, oxidase, urease, citrate). MBBL1 and MBBL2 isolates were confirmed as *E. faecalis* through a VITEK-2 system v9.01 (9). Pure colonies from MRS agar were diluted in 0.45% saline to match a 0.5 McFarland standard and then inoculated into VITEK 2 system cards. After 3 h, the system identified MBBL1 and MBBL2 isolates as *E. faecalis* based on their biochemical properties. The isolates were subcultured in nutrient broth (Biolife, Italy) overnight at 37°C, followed by DNA extraction using the QIAamp DNA Mini Kit (QIAGEN, Germany) (10). The Nextera DNA Flex library preparation kit (Illumina, San Diego, USA) was used to generate libraries from 1 ng DNA following the manufacturer’s instruction, and whole-genome sequencing was performed with an Illumina MiSeq sequencer using 2 × 250 bp protocol. Generated raw reads (MBBL1 =  2,776,552 bp; MBBL2 = 4,738,184  bp) were trimmed using Trimmomatic v0.39 (11) to remove Illumina adapters, known Illumina artifacts, and phiX reads, and quality checked using FastQC v0.12.1 (12). Reads containing Phred scores of 20 were assembled using SPAdes v3.16 (13). Genome annotation was performed using the NCBI Prokaryotic Genome Annotation Pipeline v6.6 (14) with the *Enterococcus* CheckM v1.2.2 marker set (15).

The draft genomes of MBBL1 and MBBL2 were 2,681,695 bp and 2,681,119 bp, respectively. Further sequencing and assembly statistics of both genomes are presented in Table 1. In both genomes, we identified a single antimicrobial resistance gene [*lsa*(A)], no plasmid, two prophage regions, and 242 subsystems, using ResFinder 4.0 (16), PHASTER (17), PlasmidFinder v2.1.1 (18), and RAST (19), respectively. In addition, BAGEL4 (20) and AntiSMASH 7.0 (21) revealed one bacteriocin (Enterolysin A) gene cluster and two secondary metabolite regions in both genomes. All software were run with default parameters unless specified otherwise. In conclusion, the draft genome analysis reveals the probiotic potential of *E. faecalis* strains, offering avenues for producing bioactive compounds to mitigate diseases and prompting further research.

**TABLE 1:** Genomic features of the *E. faecalis* strains isolated from raw milk of healthy cows

Isolate	Genome coverage (×)	Genome size(bp)	No. ofcontigs	*N_50_* value(bp)	GC content(%)	CheckMcompleteness	Coding sequences	RNA genes
MBBL1	57.41	2,681,695	14	576,907	37.7	99.66% (0.05% contamination)	2,526	62
MBBL2	99.81	2,681,119	13	576,907	37.7	99.66% (0.1% contamination)	2,523	62

## Data Availability

The whole-genome shotgun project of the *E. faecalis* strains MBBL1 and MBBL2 has been deposited in GenBank under accession JAZIFM000000000 and JAZIFN000000000. The raw sequences have been deposited to Sequence Read Archive (accessions: SRR27558665 for MMBL1 and SRR27558664 for MBBL2) under BioProject accession PRJNA1065156. The versions described in this paper are JAZIFM000000000.1 and JAZIFN000000000.1.
